# Supplementary figures and images for: Ancestry-specific and multi-ancestry genome-wide association studies of restless legs syndrome
Source: medRxiv. 2026 May 10:2026.04.28.26351960. Preprint. [Version 2] doi: 10.64898/2026.04.28.26351960 (PMC13174757; doi:10.64898/2026.04.28.26351960)

a)

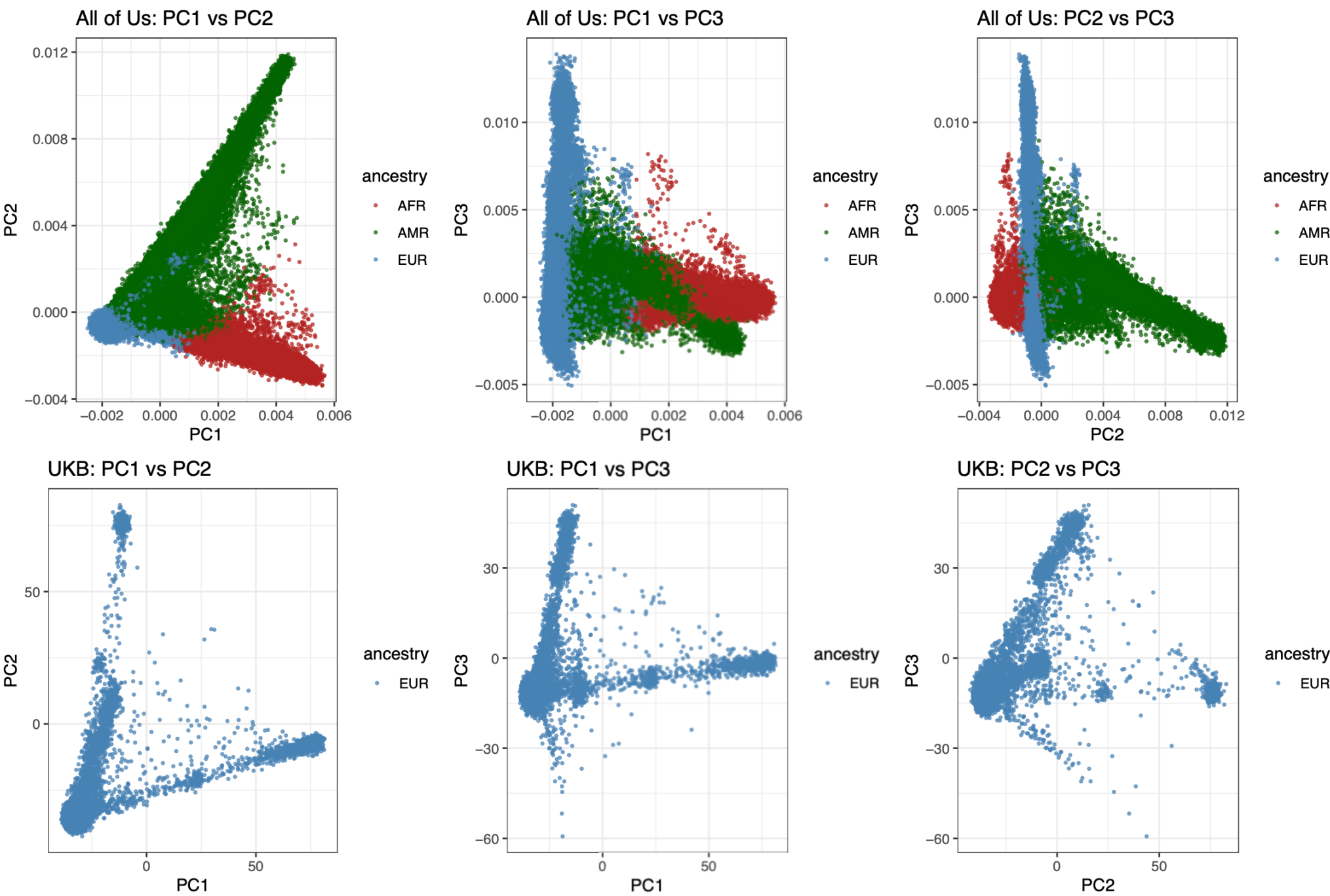

b)

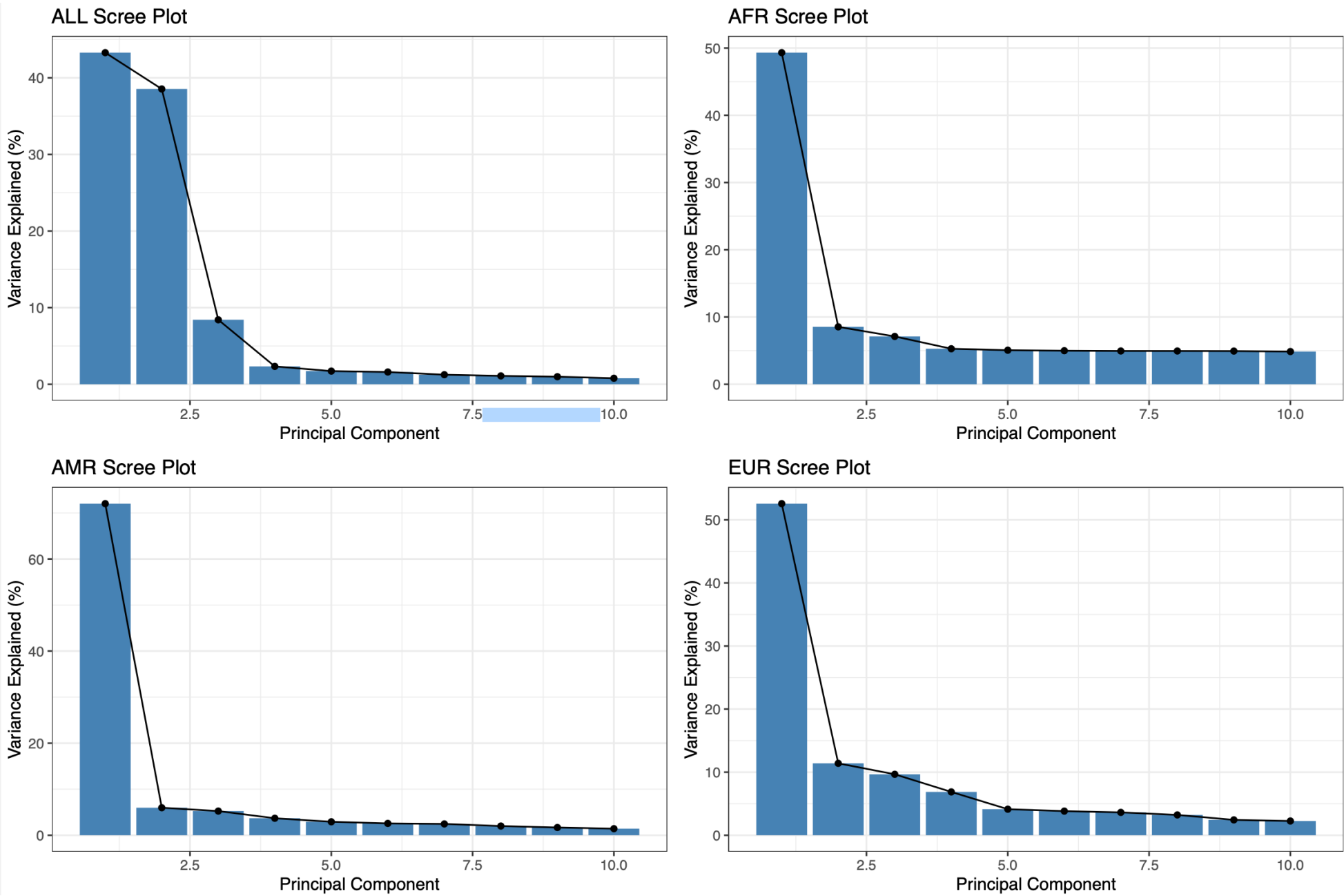

Supplement: Supplement 23 — Figure S1. Principal component structure in All of Us and UK Biobank a) Scatter plots of the first three principal components (PC1-PC3) illustrating genetic ancestry structure. b) Scree plots displaying the proportion of variance explained by the first ten principal components for the combined cohort and within each ancestry group in All of Us (AFR, AMR, EUR). [file media-23.pdf]

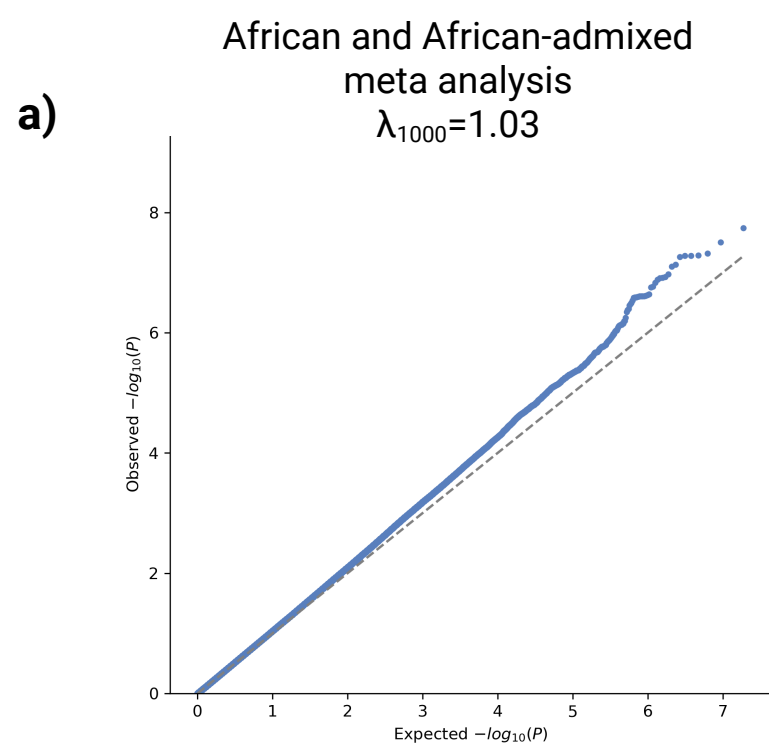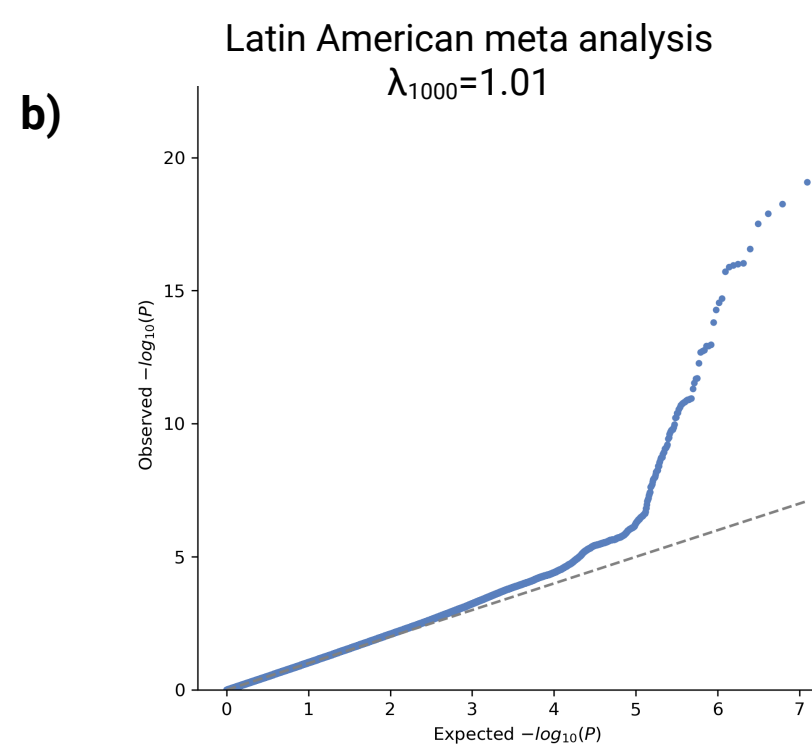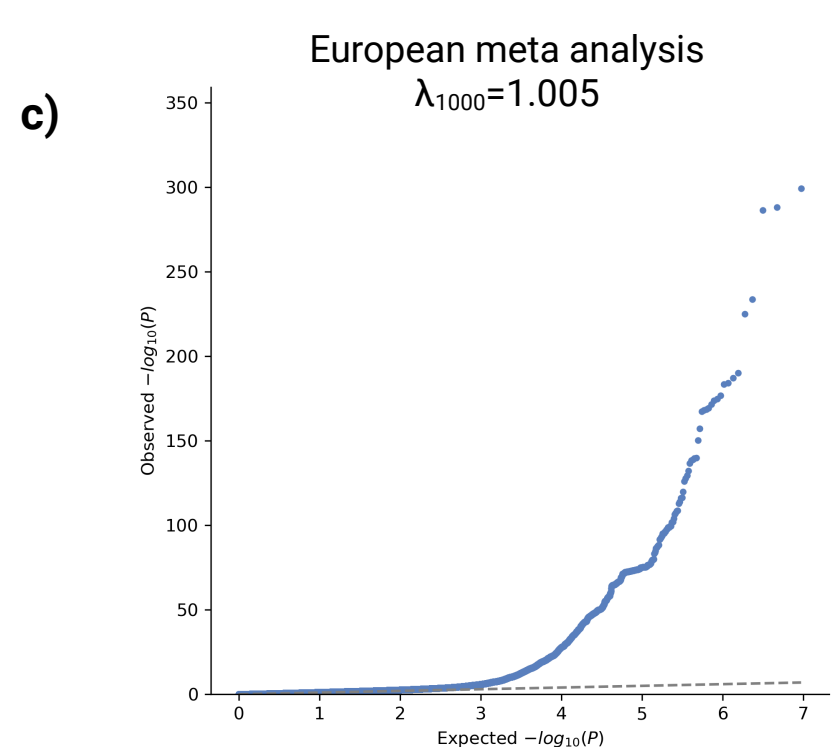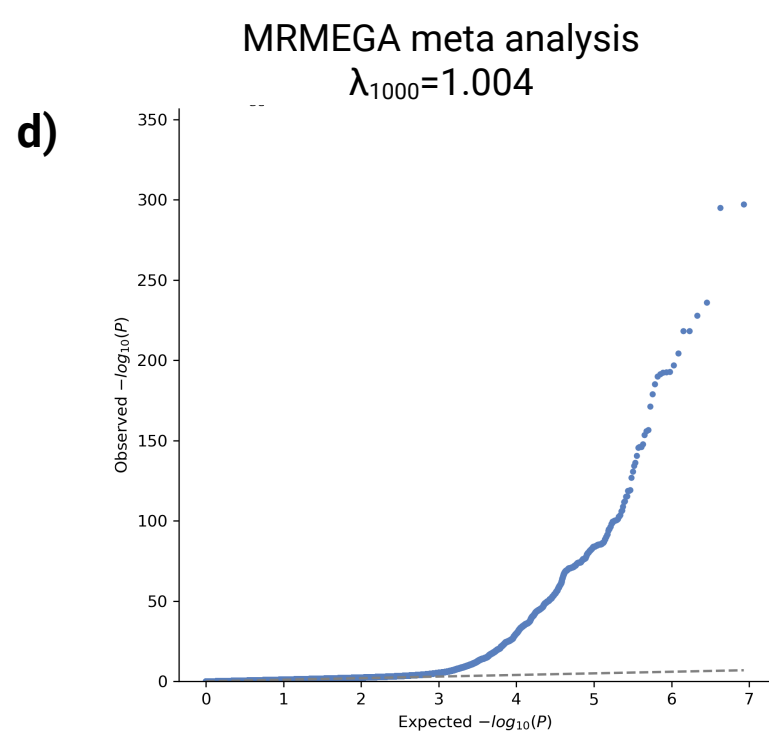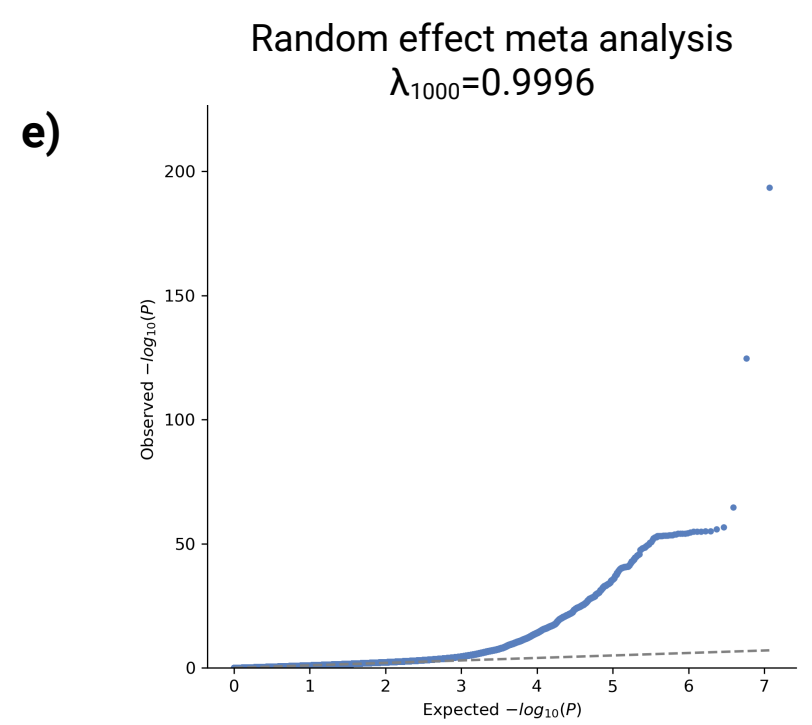

Supplement: Supplement 24 — Figure S2. Quantile-quantile (QQ) plots and genomic inflation (λ1000) for each analysis Genomic inflation values were normalized to 1000 cases and 1000 controls for all datasets to account for the large imbalance between case and control sample sizes. [file media-24.pdf]

a)

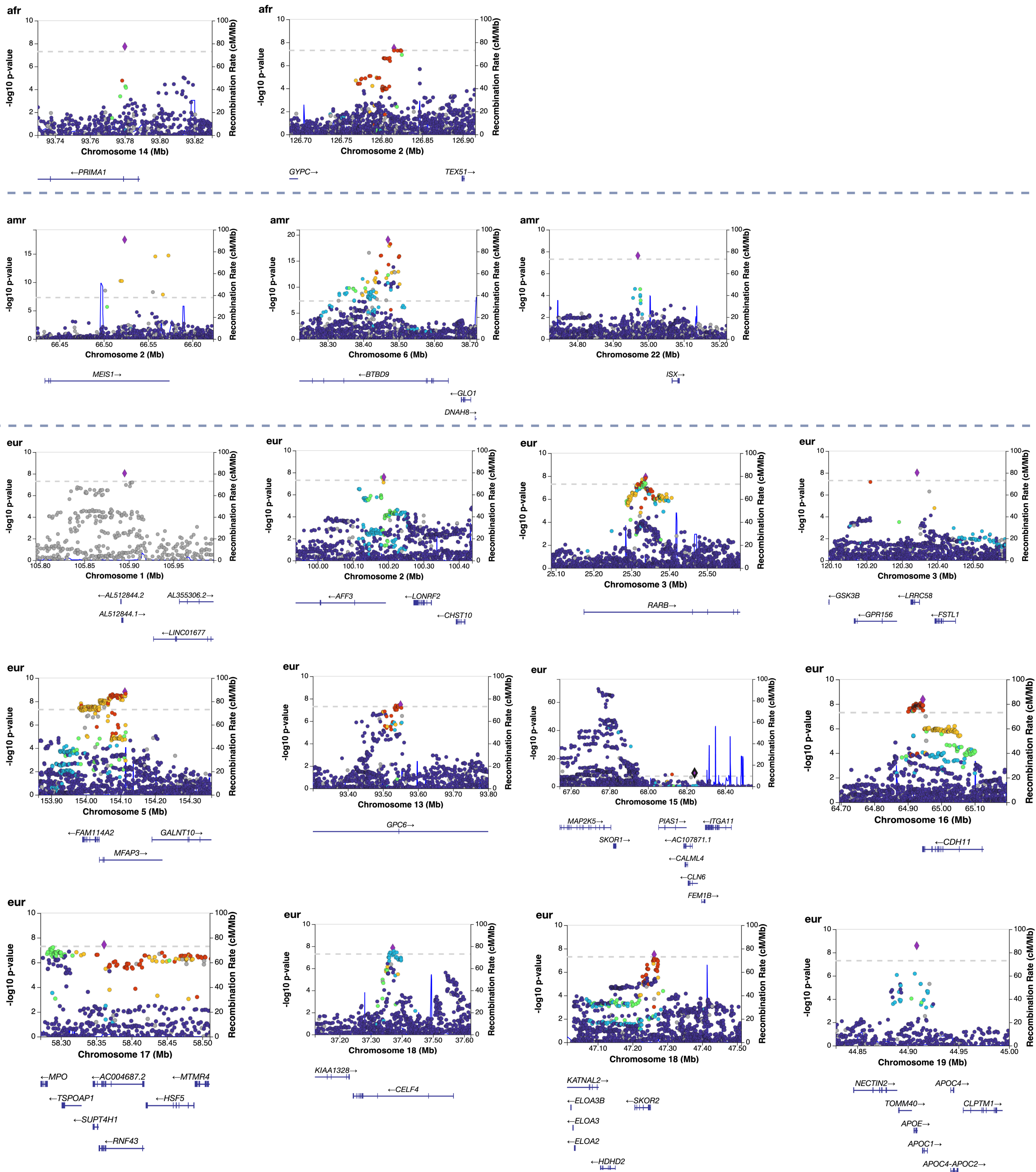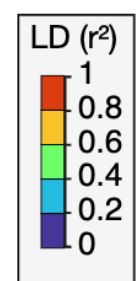

b)

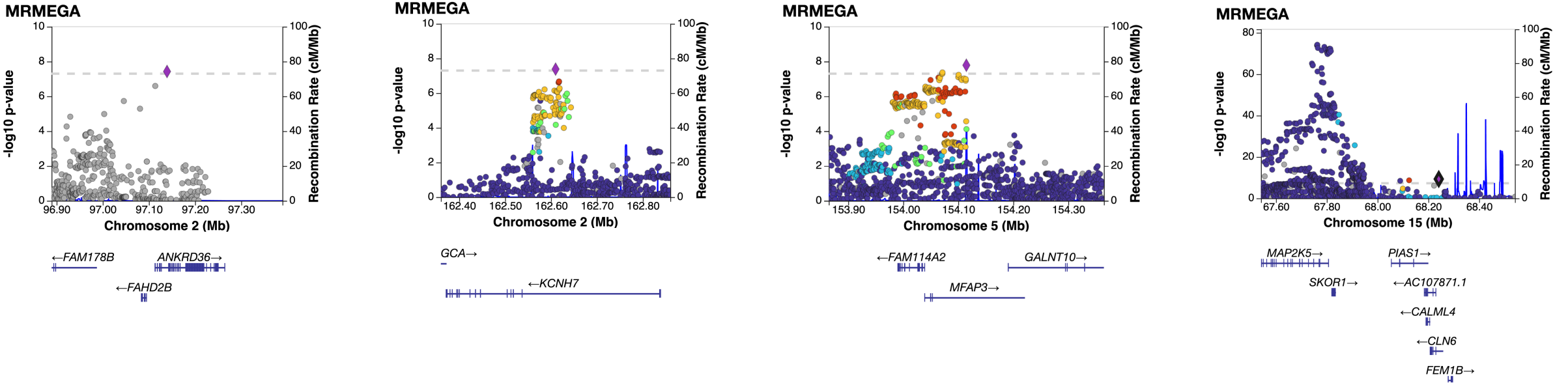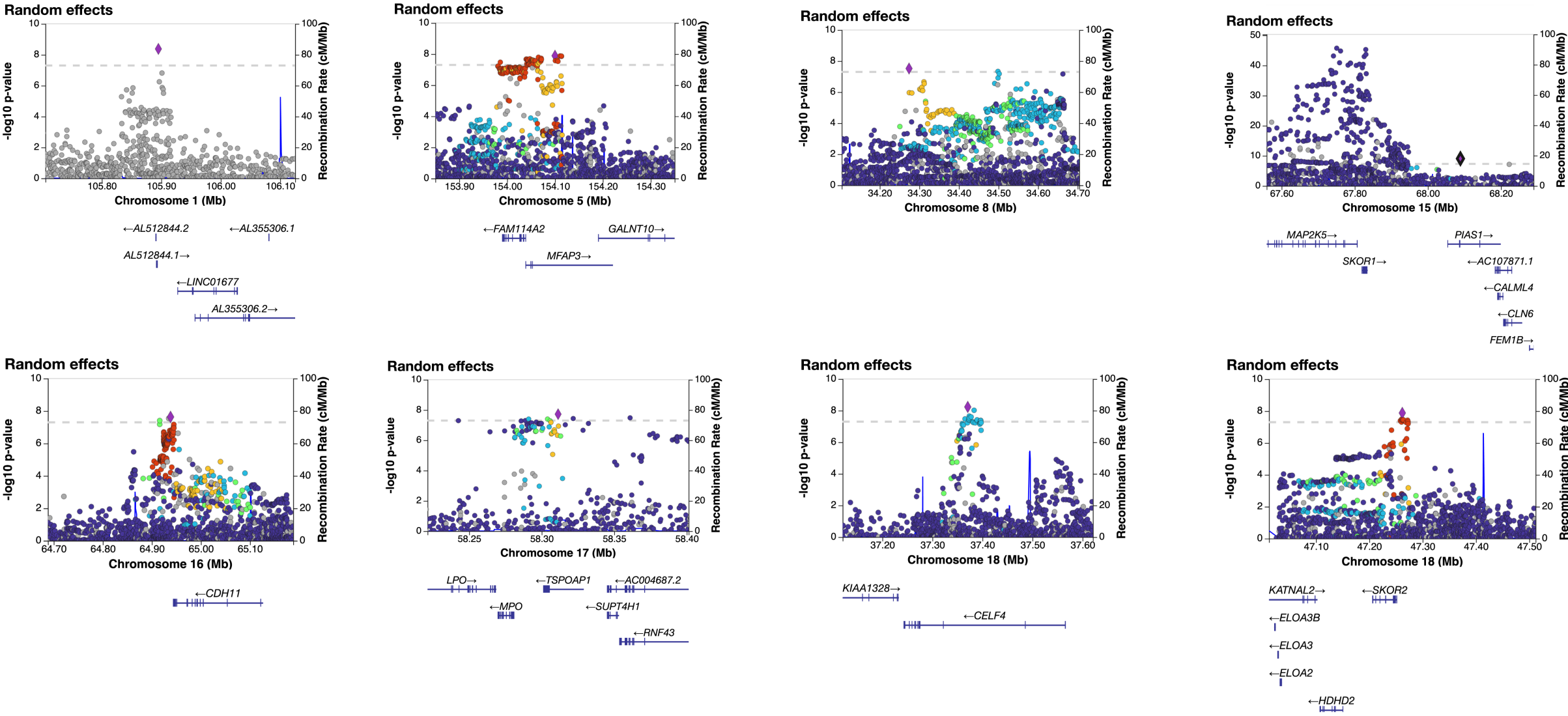

c)

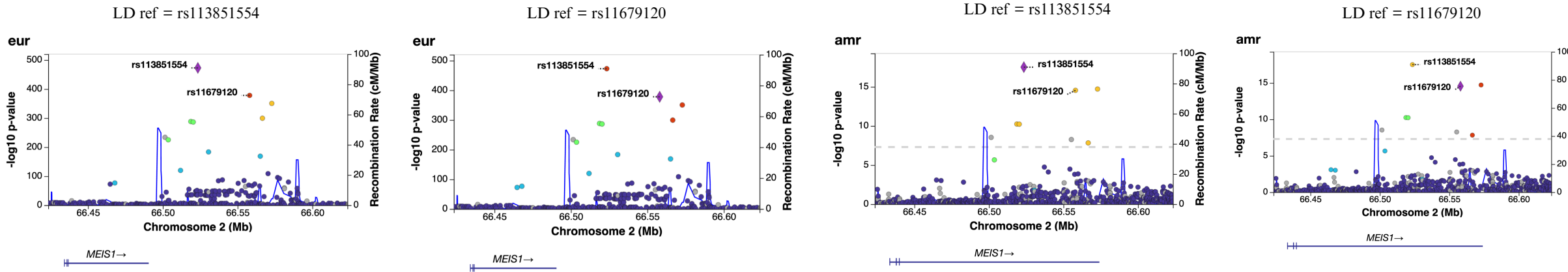

d)

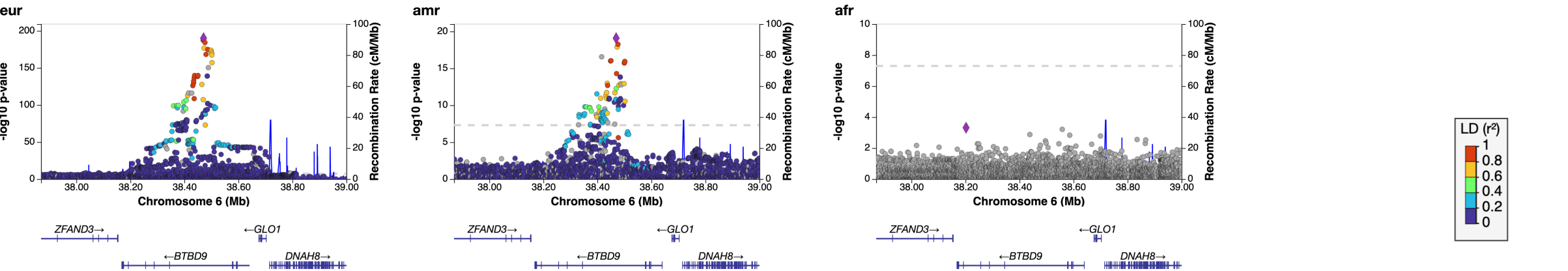

Supplement: Supplement 25 — Figure S3. a. LocusZoom plots for new loci identified in ancestry -stratified meta-analyses. b. LocusZoom plots for new loci identified in multi-ancestry MRMEGA or random effect meta-analyses. c. Regional association plots for European (EUR) and Admixed American (AMR) analyses are shown using alternative LD reference variants. d. Regional association plots for the BTBD9 locus shown across EUR, AMR, and AFR ancestry analyses. The LD color scale (bottom right) indicates the strength of linkage disequilibrium (r2) between each variant and the lead SNP, ranging from low LD (blue) to high LD (red). [file media-25.pdf]

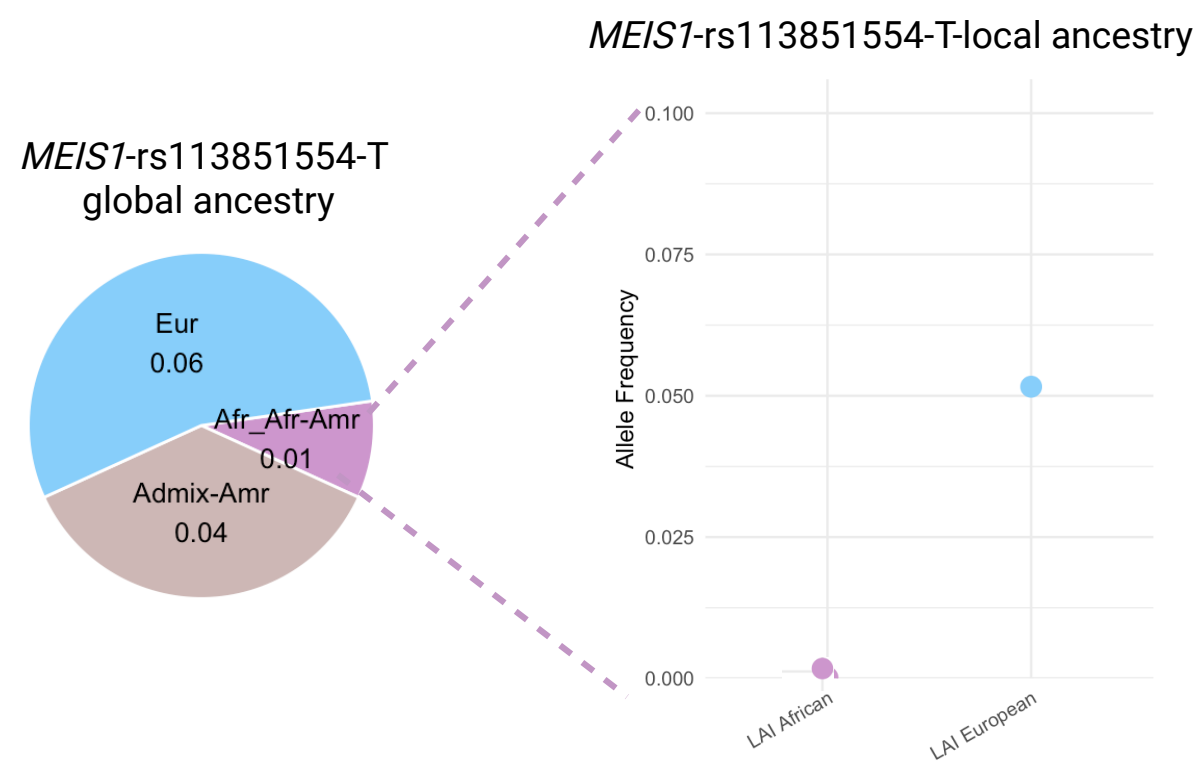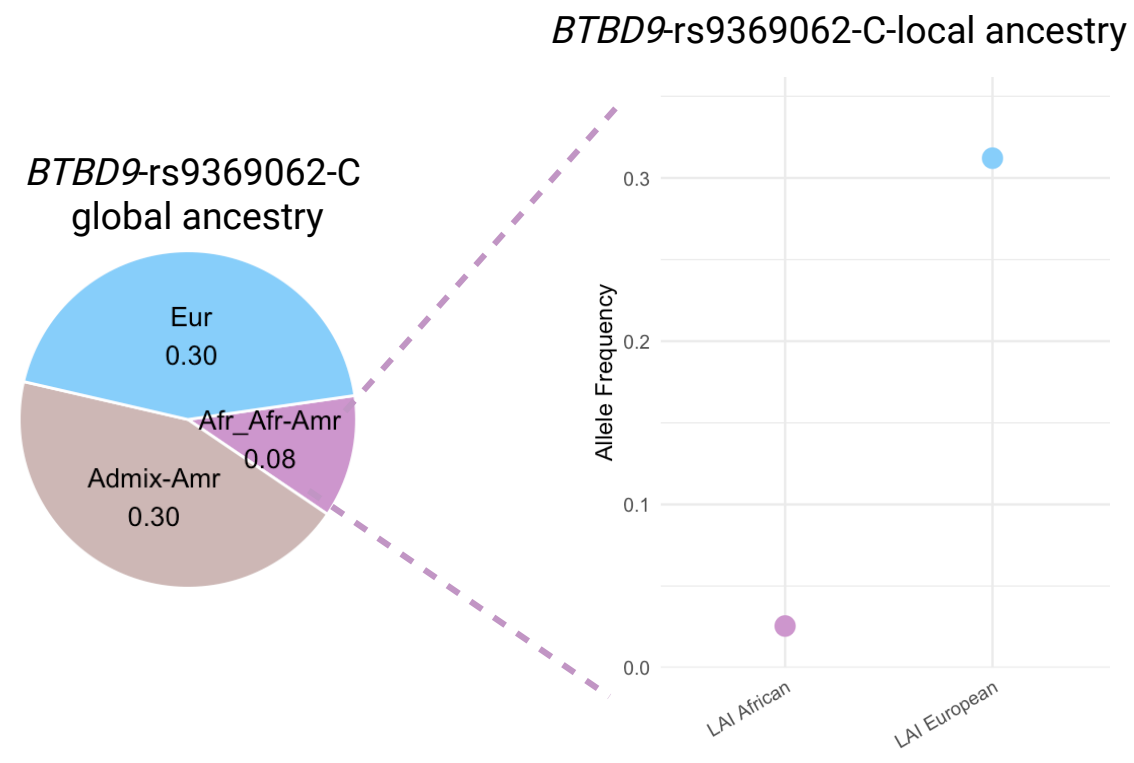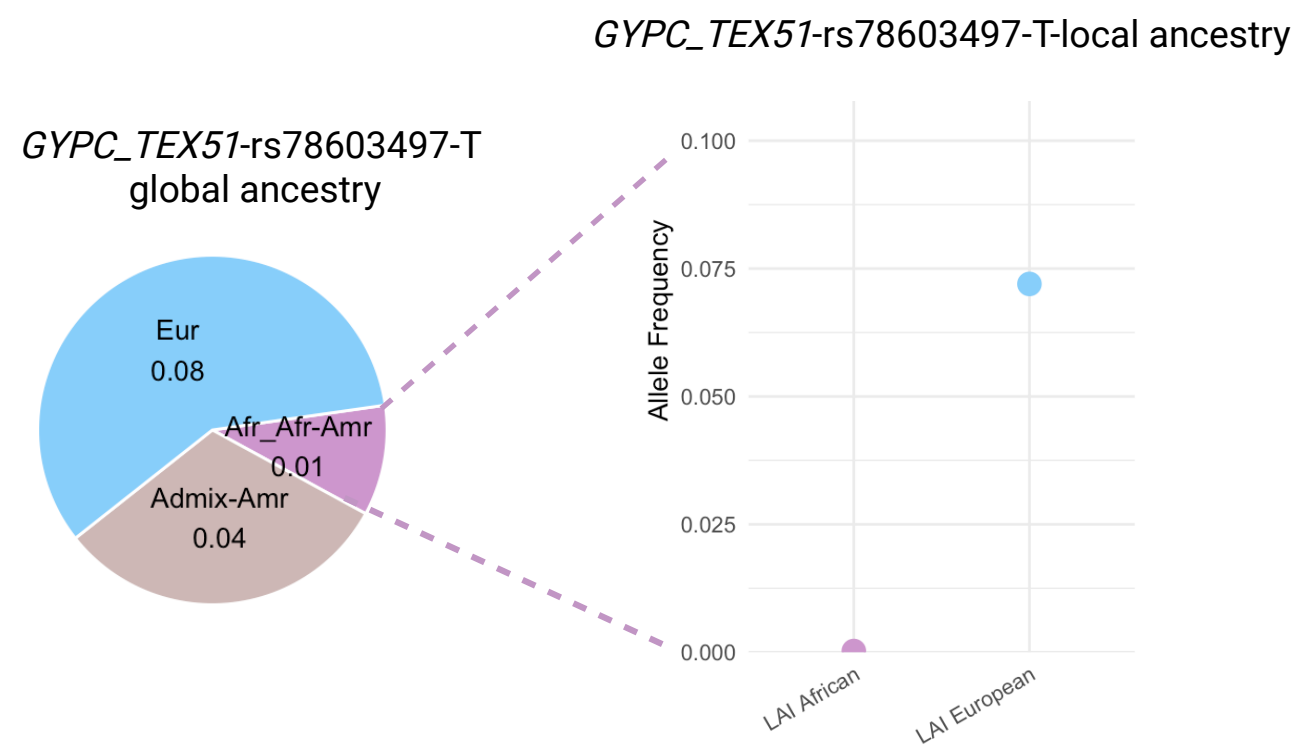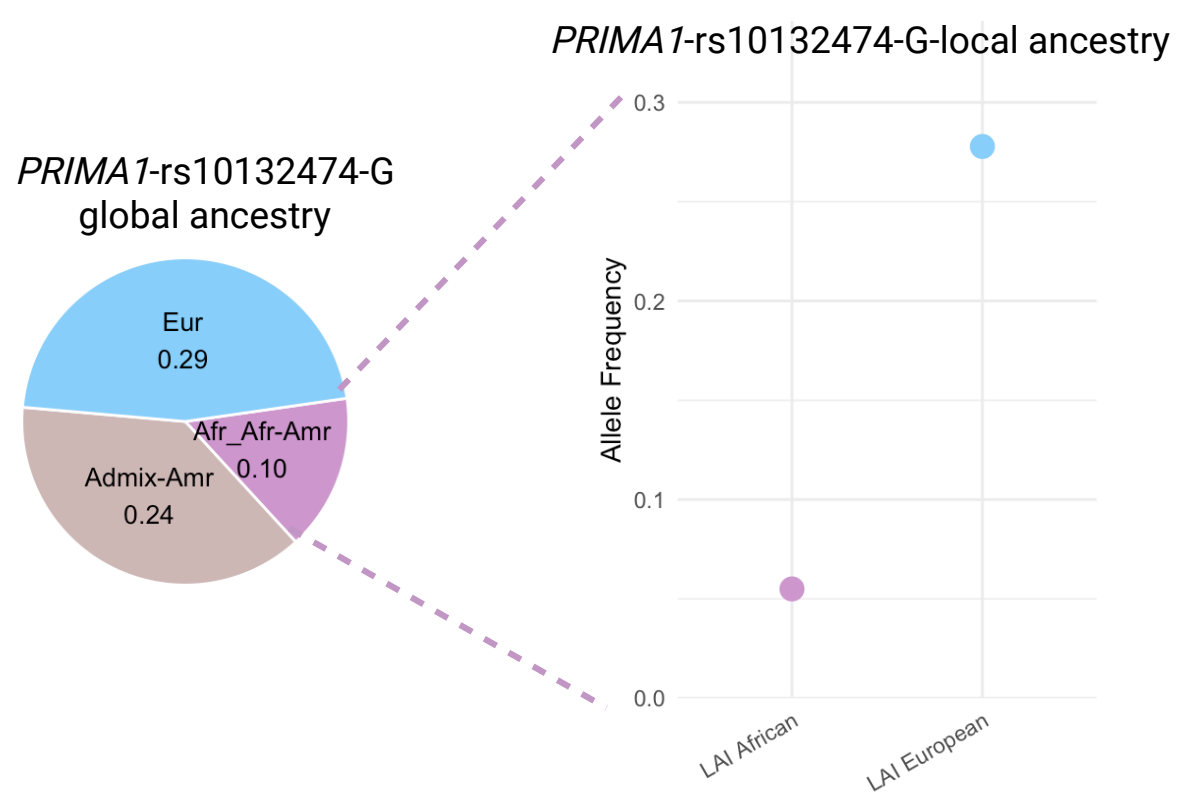

*ISX*-rs73166082-T global ancestry

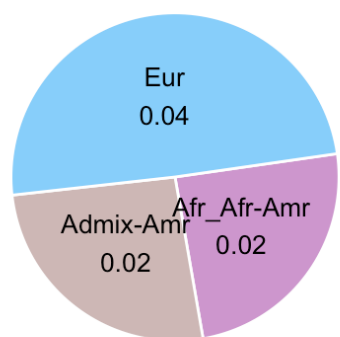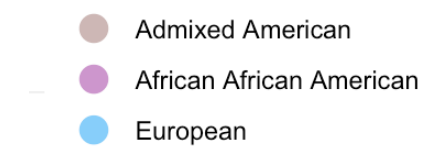

Supplement: Supplement 26 — Figure S4. Global and local ancestry allele frequency distribution of the RLS risk variants identified in African and African admixed and Latin American cohorts: MEIS1, BTBD9, GYPC/TEX51, PRIMA1, and ISX. Local ancestry inference (LAI) for African or African American and Admixed American ancestry groups was obtained from gnomAD v4.1. LAI for the ISX lead variant was unavailable. [file media-26.pdf]

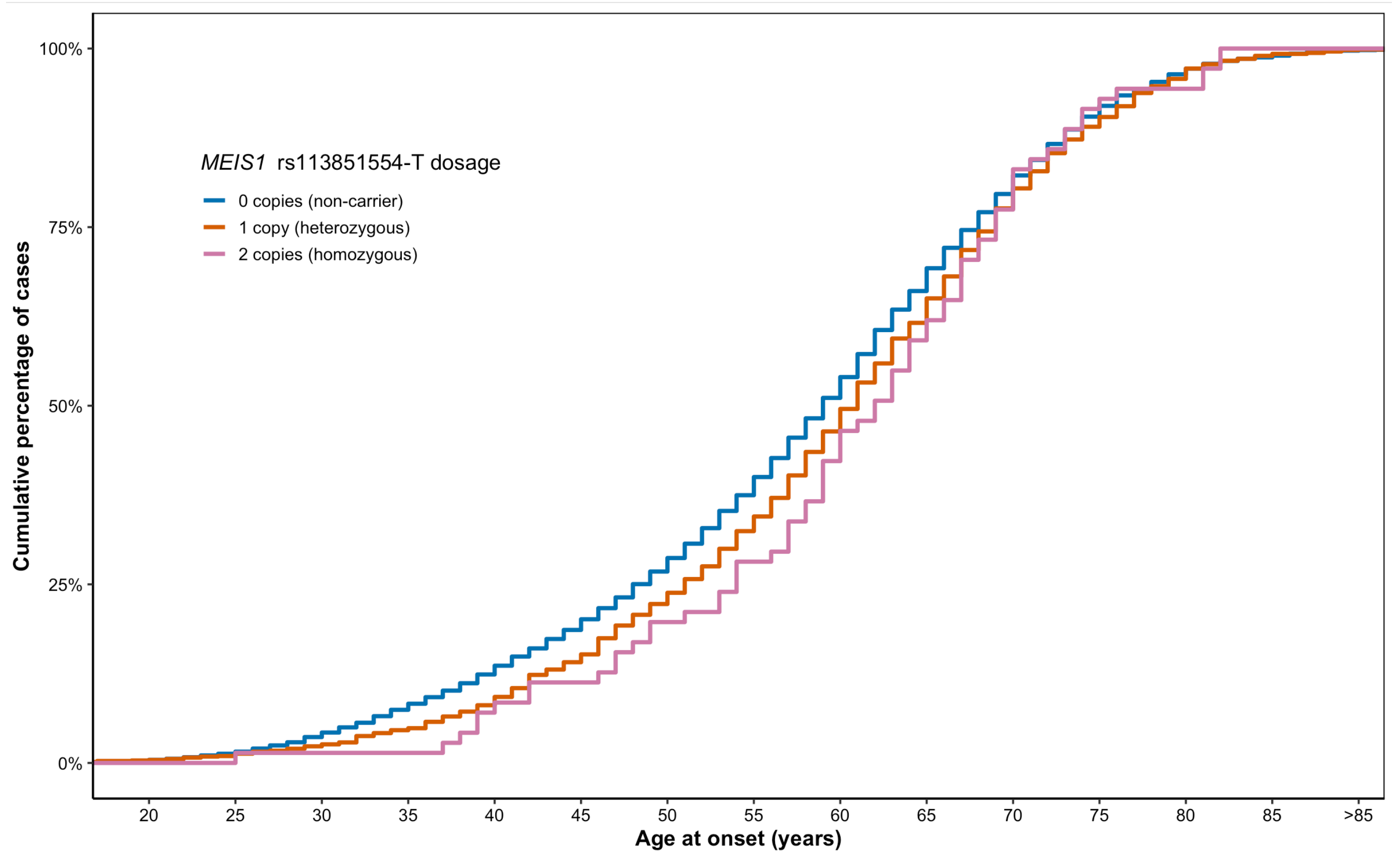

Supplement: Supplement 27 — Figure S5. Age at onset of restless legs syndrome according to MEIS1 rs113851554 genotype. The cumulative distribution of age at onset is shown for RLS cases grouped by rs113851554 genotype dosage (0, 1, or 2 copies of the T allele). Age at onset analyses were performed in RLS cases from the All of Us cohort using linear regression adjusted for sex and the first ten principal components. The rs113851554 T allele was associated with a later age at onset (β = 1.54 years per allele, SE = 0.36; p = 2.03 × 10^−5^). [file media-27.pdf]

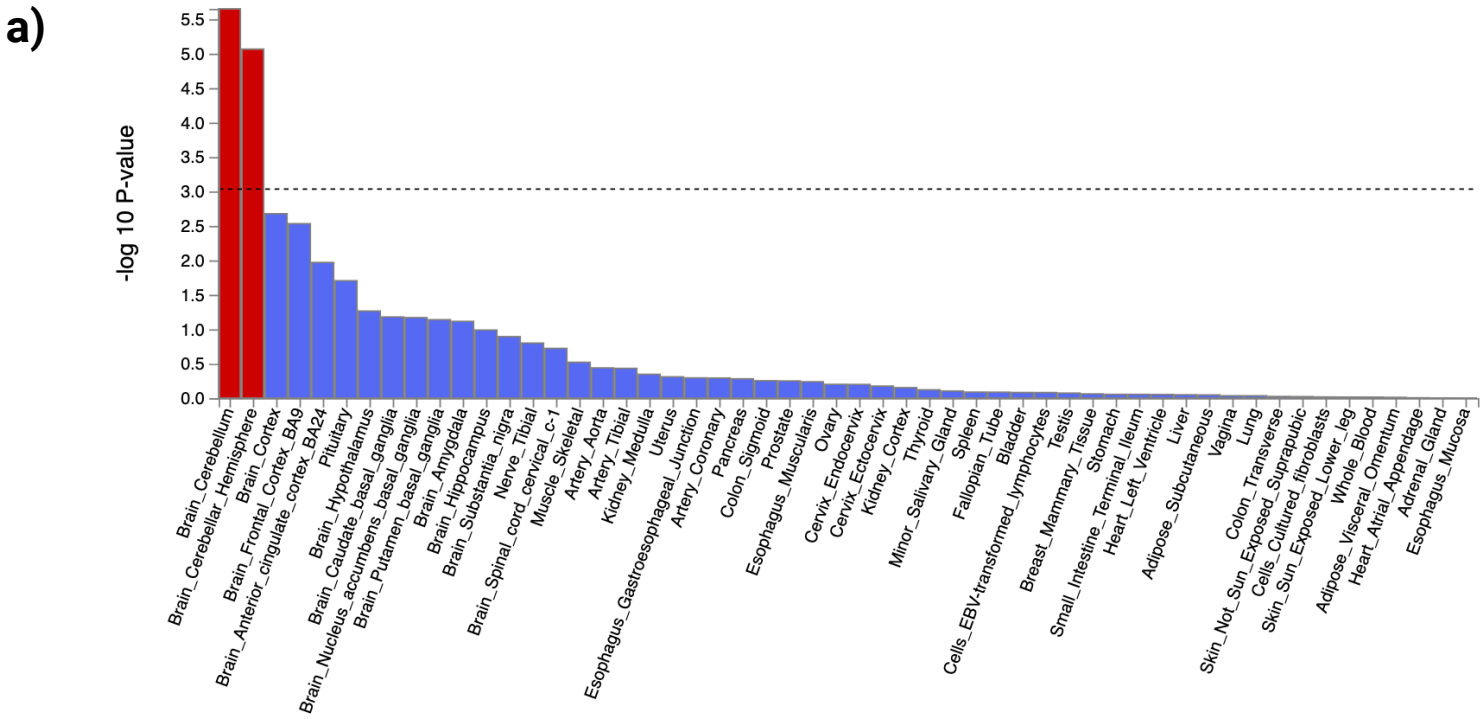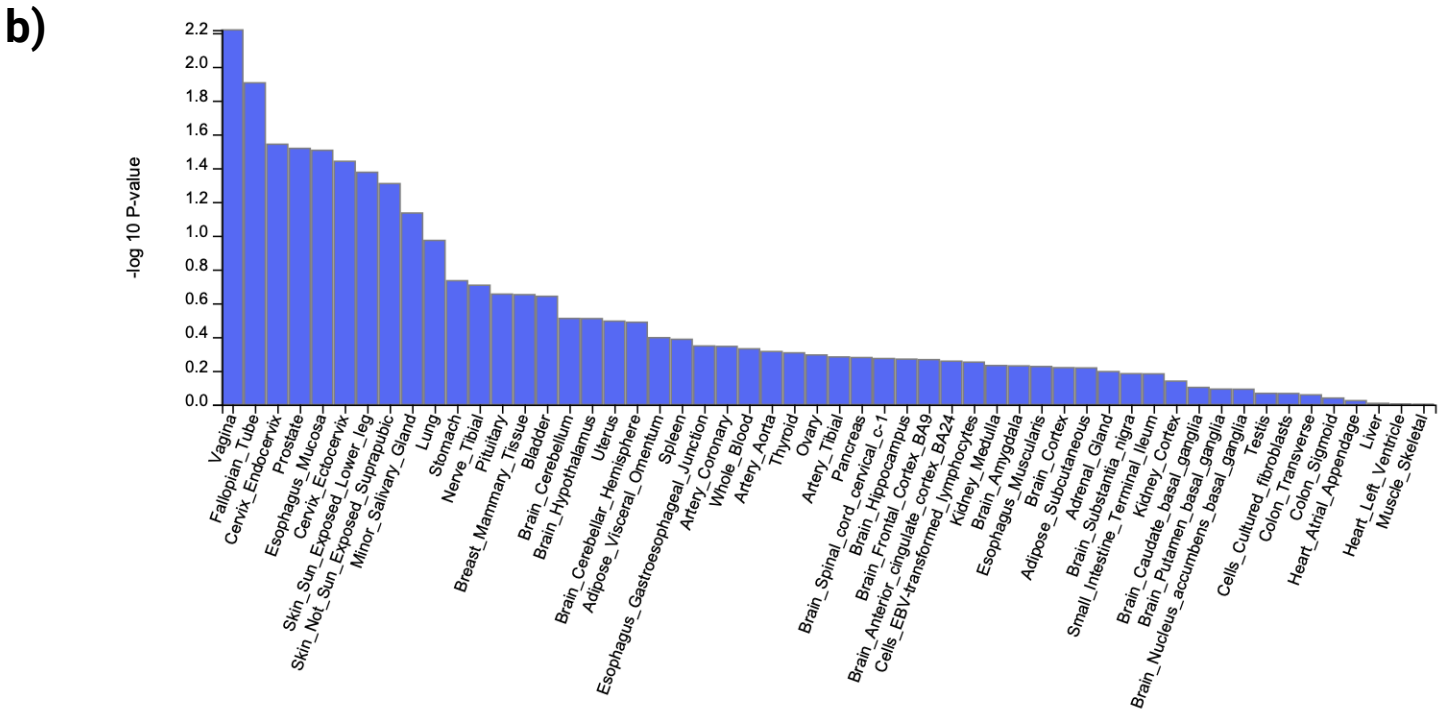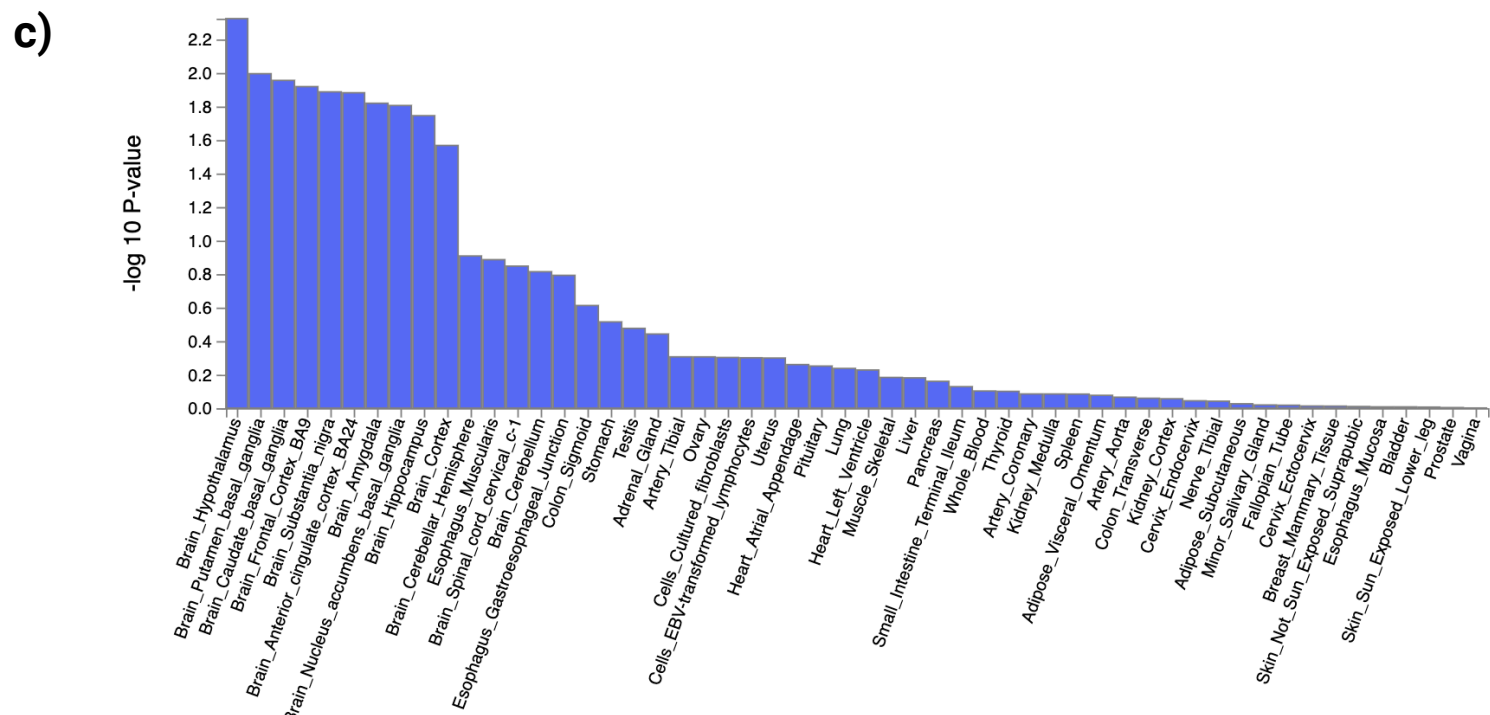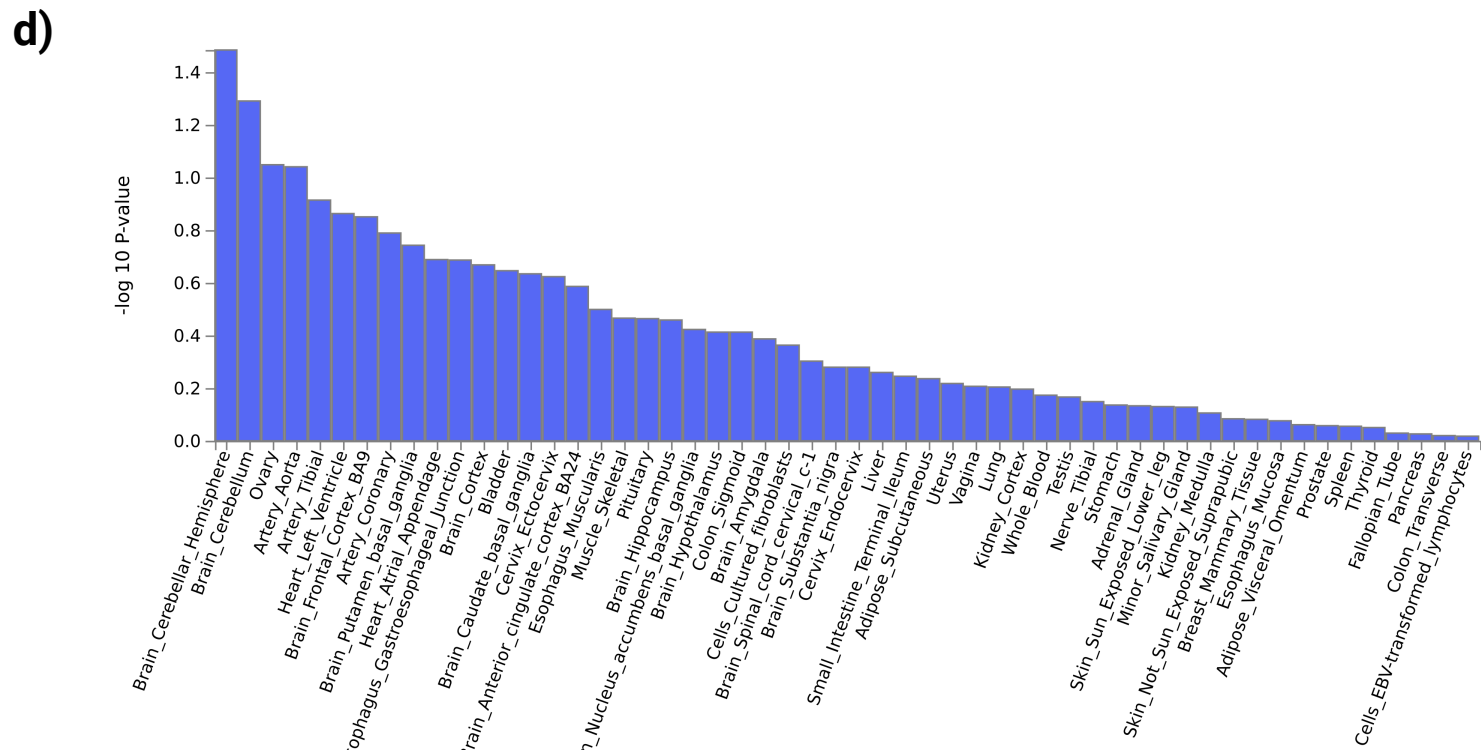

Supplement: Supplement 28 — Figure S6. Tissue-specific expression enrichment of RLS-associated genes based on MAGMA analysis. [file media-28.pdf]

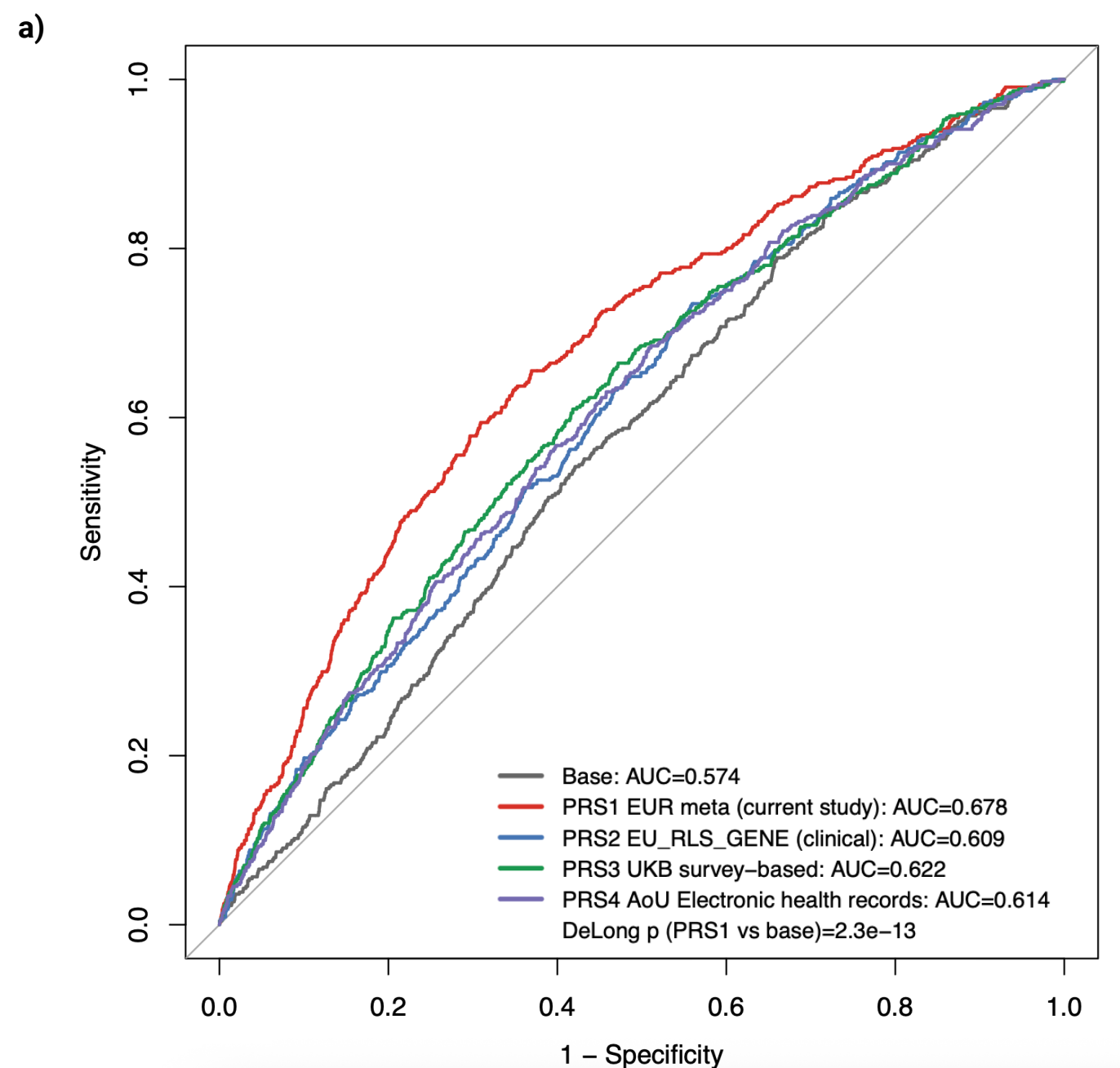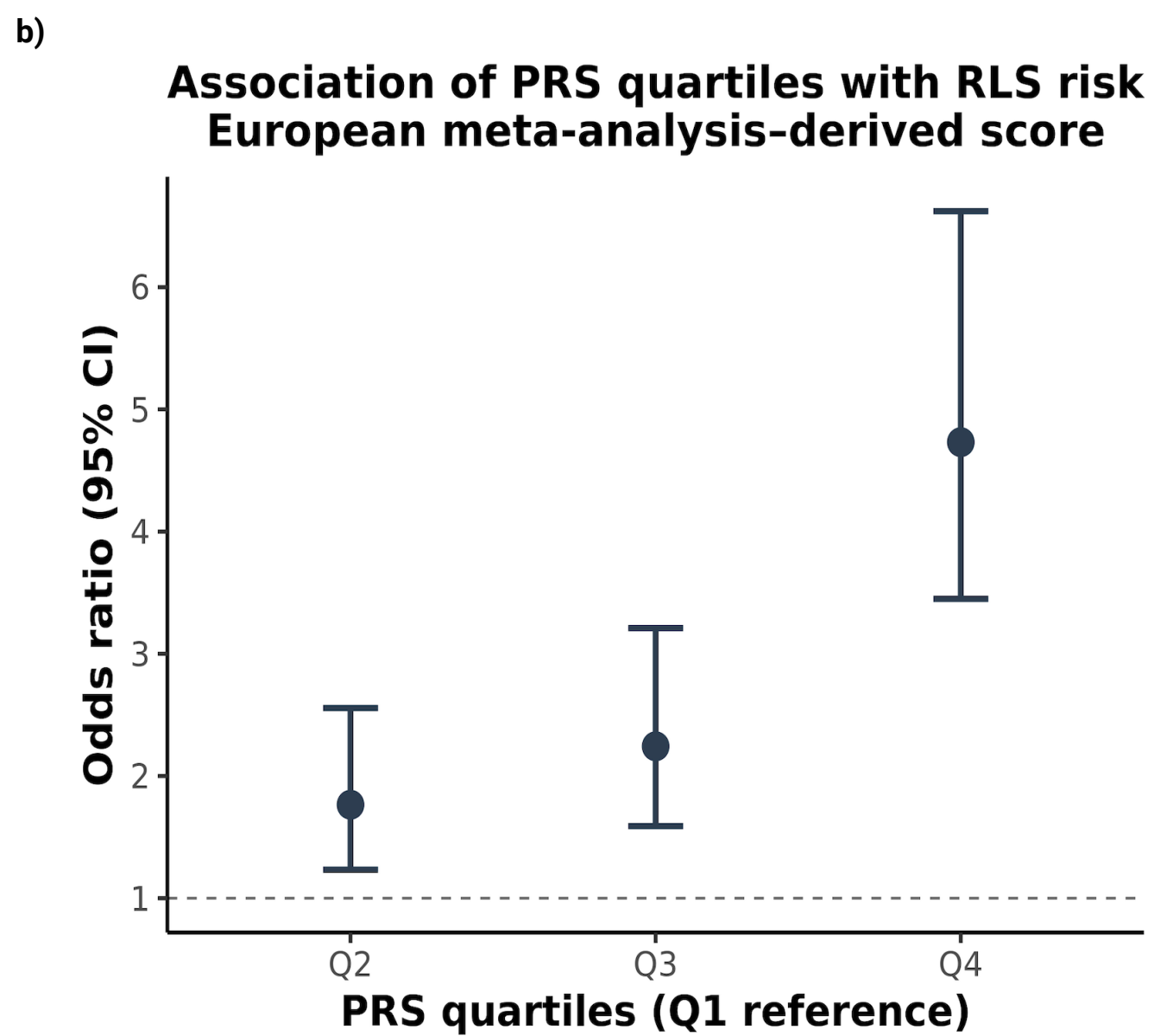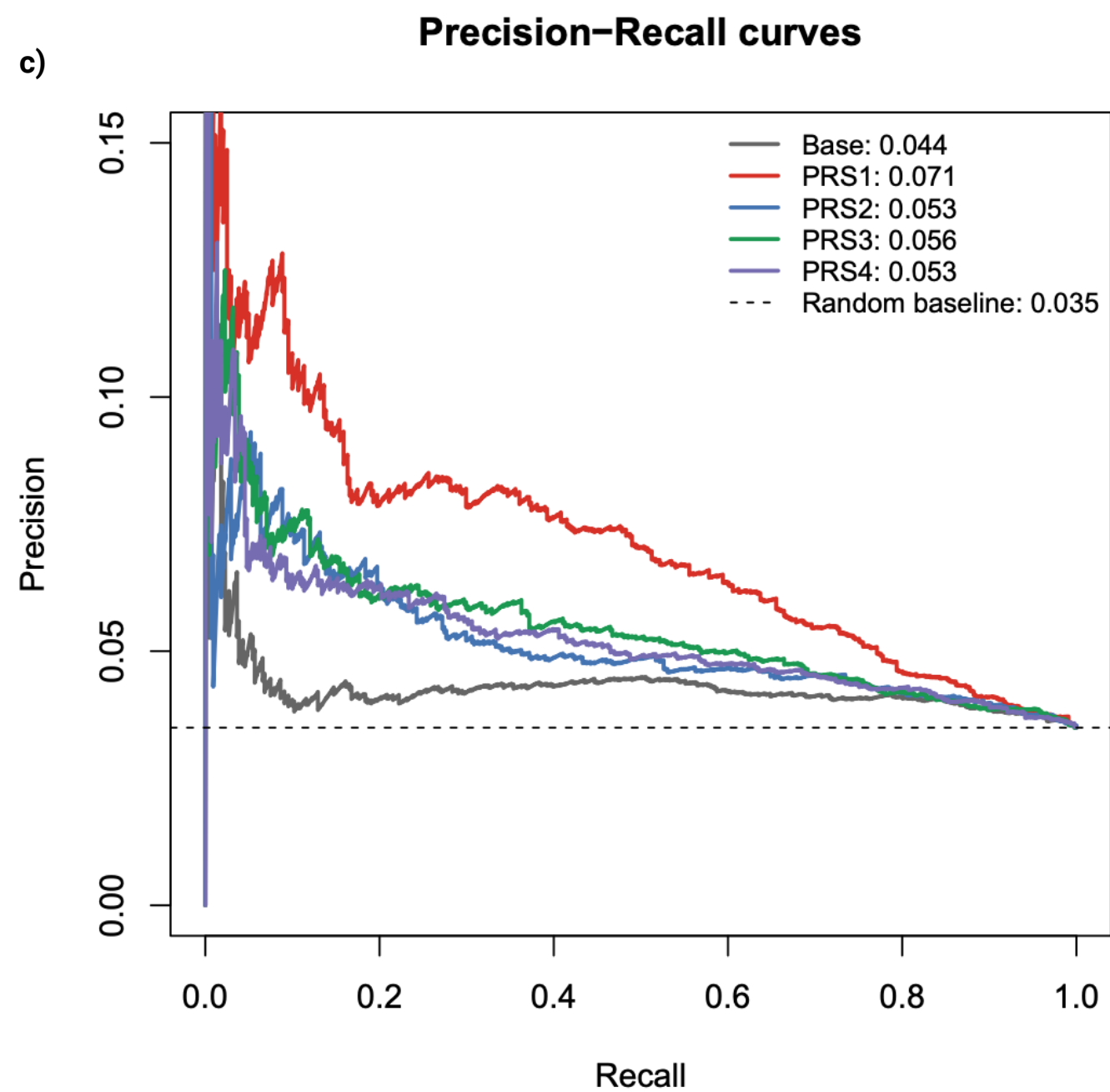

Supplement: Supplement 29 — Figure S7. Performance of polygenic risk scores for RLS in an independent cohort. a) Receiver operating characteristic curves comparing base clinical model (age, sex, PCs) with PRS derived from four training GWAS datasets: European multi-cohort meta-analysis, EU-RLS-GENE (clinically ascertained), UK Biobank survey-based, and All of Us EHR-based. b) Association of European meta-analysis-derived PRS quartiles with RLS risk. Odds ratios were estimated using logistic regression adjusted for age, sex, and first ten PCs; Q1 used as reference. c) Precision-recall curves for the same models. The dashed horizontal line indicates the case prevalence in the target cohort, representing random classifier performance. [file media-29.pdf]
